# Role of Fibronectin in Primary Open Angle Glaucoma

**DOI:** 10.3390/cells8121518

**Published:** 2019-11-26

**Authors:** Jennifer A. Faralli, Mark S. Filla, Donna M. Peters

**Affiliations:** 1 Departments of Pathology & Laboratory Medicine, University of Wisconsin, Madison, WI 53706, USA; peters10@wisc.edu (J.A.F.); msfilla@wisc.edu (M.S.F.); 2 Ophthalmology & Visual Sciences, University of Wisconsin, Madison, WI 53706, USA

**Keywords:** trabecular meshwork, integrin, fibronectin, Schlemm’s canal, glaucoma

## Abstract

Primary open angle glaucoma (POAG) is the most common form of glaucoma and the 2nd most common cause of irreversible vision loss in the United States. Nearly 67 million people have the disease worldwide including >3 million in the United States. A major risk factor for POAG is an elevation in intraocular pressure (IOP). The increase in IOP is believed to be caused by an increase in the deposition of extracellular matrix proteins, in particular fibronectin, in a region of the eye known as the trabecular meshwork (TM). How fibronectin contributes to the increase in IOP is not well understood. The increased density of fibronectin fibrils is thought to increase IOP by altering the compliance of the trabecular meshwork. Recent studies, however, also suggest that the composition and organization of fibronectin fibrils would affect IOP by changing the cell-matrix signaling events that control the functional properties of the cells in the trabecular meshwork. In this article, we will discuss how changes in the properties of fibronectin and fibronectin fibrils could contribute to the regulation of IOP.

## 1. Introduction to Glaucoma

**Figure 1:**
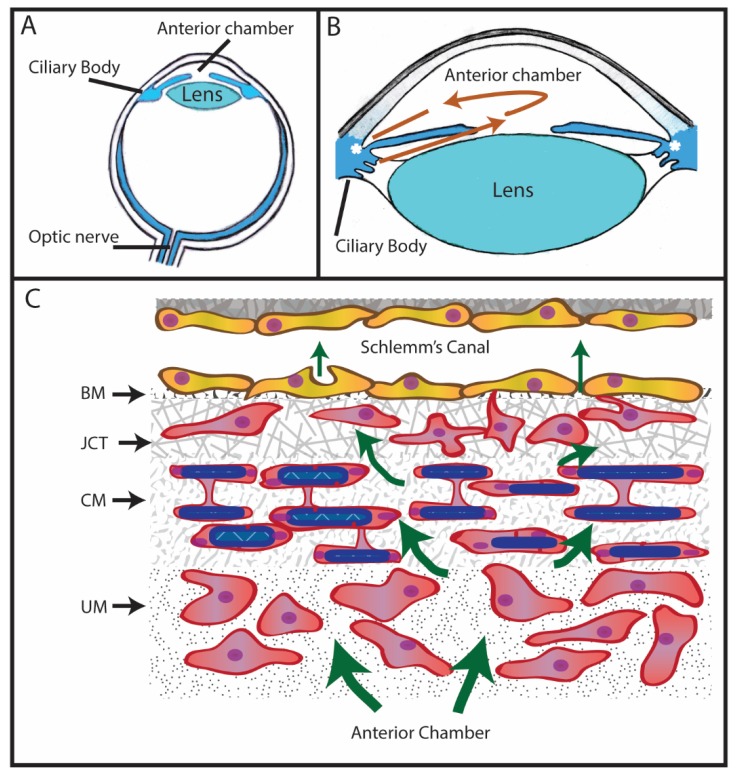
Anatomy of the eye and trabecular meshwork: (**A**) Eye diagram showing anterior chamber between the iris and the cornea, the ciliary body that produces aqueous humor, the lens, and the optic nerve. (**B**) Aqueous humor (red arrows) produced by the ciliary body flows between the lens and iris into the anterior chamber before leaving the eye through the trabecular meshwork (asterisk). (**C**) Schematic of the trabecular meshwork and Schlemm’s canal: Aqueous humor flows from the anterior chamber through the 3 layers of the trabecular meshwork called uveoscleral meshwork (UM), corneoscleral meshwork (CM), and the juxtacanalicular tissue (JCT). Aqueous humor (arrow) then crosses the basement membrane (BM) of Schlemm’s Canal either paracellularly or transcellularly and enters into Schlemm’s Canal.

Glaucoma is an age-related, heterogeneous group of neurodegenerative diseases that ultimately results in damage to the optic nerve and irreversible blindness. Primary open angle glaucoma (POAG) is one form of glaucoma and is the most common form found in the United States [1,2]. It results from damage to the optic nerve, which is believed to be due to a chronic elevation in intraocular pressure (IOP). IOP is regulated by the level of aqueous humor found in the anterior chamber of the eye (Figure 1). Aqueous humor is produced by the epithelium of the ciliary body and is responsible for providing nutrients to the avascular ocular tissues found in the front of the eye, including the cornea, the trabecular meshwork, and the lens. In a normal eye, it enters into the anterior chamber of the eye through the pupil and then slowly drains at approximately 2.75 μL/min [3] out of the anterior chamber through the region of the anterior segment called the trabecular meshwork (TM). The TM is located at the angle formed where the iris meets the cornea around the circumference of the eye (Figure 1B). As shown in Figure 1C, this region is made up of several distinct cell layers. They are the uveal meshwork, corneoscleral meshwork, the juxtacanalicular tissue (JCT), and the endothelial lining of Schlemm’s canal (SC). From the TM, aqueous humor exits into Schlemm’s Canal and eventually enters circulation [3]. IOP is determined by the level of aqueous humor secreted from the epithelium of the ciliary body and by drainage of it through the TM/SC.

In POAG, there is an imbalance between the production and drainage of aqueous humor within the anterior segment that results in an elevation of IOP. This imbalance is often the result of a restriction in the movement of aqueous humor through the TM/SC outflow pathway. Most of the resistance to aqueous humor outflow is thought to be within the deepest portion of the TM consisting of the JCT and the basement membrane beneath the inner endothelium wall of Schlemm’s canal [4,5].

What generates and regulates the resistance in the JCT is not well understood. In the normal eye, outflow resistance in this region would be lowered through relaxation of the contractile fibroblastic-like cells in the JCT and the adjacent sclera. In addition, the contraction of the ciliary muscle which is found within the ciliary body and is connected to the JCT via an elastin network would result in a change in geometry of the TM/SC that widens the outflow pathway and decreases the resistance [6]. However, in POAG, there is a buildup of extracellular matrix (ECM) proteins within the JCT, especially fibronectin, which is believed to contribute to the increase in resistance to aqueous humor outflow through the TM/SC. Such alterations in the expression of ECM proteins are thought to lead to changes in the biological characteristics of resident cells in the JCT, which more and more acquire the structural and functional characteristics of contractile myofibroblasts [6,7,8]. These changes to the contractility of the actomyosin network are likely to be transmitted by the surrounding ECM via integrins.

## 2. Expression of Fibronectin in the Trabecular Meshwork

Fibronectin is one of the major ECM proteins in the TM [9]. It is found in the sheath material surrounding the elastin tendons that enter the TM from the ciliary muscle within the ciliary body. These tendons are connected to a network of elastin fibers in the JCT that are connected to the inner wall of Schlemm’s canal and help contribute to the contraction and relaxation of the TM/SC. Fibronectin is also present in the amorphous fibrogranular material distributed throughout the JCT. Additionally, it is found scattered throughout the basement membranes underneath the cells lining the outside of the trabecular beams and in the discontinuous basement membrane underneath the cells lining the inner wall of Schlemm’s canal. Additionally, fibronectin is found in the core of the trabecular beams and as a soluble protein in aqueous humor [10].

During aging and in some patients with POAG, increases in fibronectin expression have been observed in the TM/SC, especially in the sheath material along the elastin tendons, which is therefore likely to effect the functionality of the elastin network in the TM/SC [9,11,12,13]. Increased levels of fibronectin have also been found in the JCT in some patients with POAG and in aqueous humor obtained from patients with glaucoma. Studies comparing the levels of fibronectin in aqueous humor found in patients with cataracts to that of glaucoma patients showed that the levels of fibronectin were nearly sevenfold higher in glaucomatous patients (0.136 ± 0.192 µg/mL vs. 0.962 ± 0.918 µg/mL, respectively) [14,15].

The cause for the increase in fibronectin expression in glaucoma is unknown, but it may be due to the elevated levels of transforming growth factor-β2 (TGF-β2) found in aqueous humor in approximately 50% of patients with POAG [16,17,18,19]. In normal eyes, the total amount of TGF-β2 in aqueous humor ranged from 0.41 to 2.24 ng/mL and approximately 37% was in the active form. In contrast, the level of TGF-β2 in aqueous humor from POAG patients ranged from 1.4 to 2.70 ng/mL and 60.84% of the total amount was active [16,17]. Numerous studies have now shown that TGF-β2 leads to an elevation in fibronectin expression in both cultured TM cells in vitro as well as in vivo [20,21,22]. Sources for the high amounts of TGF-β2 in aqueous humor are probably the epithelial layers of the ciliary body [23] and the lens [24,25,26]. However, TM cells in culture have also been shown to produce TGF-β2 [16] as well as to express TGF-β receptors [27].

The action of TGF-β2 in POAG and on fibronectin expression is largely mediated through connective tissue growth factor (CTGF). CTGF is constitutively expressed in the TM in situ [28] and in cultured TM cells treated with TGF-β2 [29,30]. Studies showing that silencing CTGF expression in TM cells in culture prevents the TGF-β2-induced increase in fibronectin synthesis and supports the idea that CTGF is a key mediator of fibronectin expression in the TM [31]. In addition, transgenic mice overexpressing CTGF in their eyes exhibit an elevation in IOP that correlates with the loss of optic nerve axons typically observed in POAG [32].

The result of TGF-β2 signaling is not restricted to increasing fibronectin synthesis. TGF-β2 also increases the expression and activity of an enzyme called tissue transglutaminase [33,34], which induces irreversible cross-linking of fibronectin in the ECM [34]. The increase in cross-linked fibronectin could contribute to the increased aqueous humor outflow resistance observed in POAG [35].

Another observation that suggests that fibronectin expression in the TM may be involved in glaucoma is that approximately 40% of the general population treated with glucocorticoids, such as dexamethasone, develop an elevated IOP [36,37] and that these tissues exhibit an increase in fibronectin synthesis [22,38,39,40]. A secondary glaucoma called glucocorticoid-induced glaucoma can develop in 3–8% of the glucocorticoid responder population. Clinically, POAG is very similar to glucocorticoid-induced glaucoma [38]. Both exhibit similar increases in fibronectin and ECM protein expression, a restriction in aqueous humor outflow, and an elevation in IOP. Also, nearly all POAG patients respond to glucocorticoids with an increase in IOP [7,38,41,42].

Furthermore, inhibiting fibronectin expression prevents the ER stress response that is observed in cultured human TM cells treated with dexamethasone and associated with a mouse model of glucocorticoid-induced glaucoma [43]. Conversely, overexpression of an isoform of fibronectin containing the extra domain A (EDA) induced chronic ER stress in human TM cell cultures [44]. Glucocorticoids have also recently been shown to cause an elevation in TGF-β2 in cultured TM cells and in the aqueous humor of a mouse model of glucocorticoid-induced glaucoma [45]. These observations support the idea that fibronectin may be involved in POAG since TGF-β2 is a key regulator of fibronectin synthesis.

Finally, the upregulation of fibronectin in response to glucocorticoids may trigger certain changes within TM cells that are associated with POAG. For example, formation of cross-linked actin networks (CLANs) is associated with POAG and glucocorticoid-induced glaucoma and fibronectin has been shown to trigger CLAN formation in cultured TM cells [46]. Similarly, when TM cells are exposed to excess fibronectin within the ECM, expression of proteins associated with ER stress has been demonstrated [44].

Paradoxically, angiopoietin-like 7 (ANGPTL7) expression, which is upregulated by dexamethasone or TGF-β2 [47,48,49,50,51], has the opposite effect on fibronectin expression and fibronectin fibril formation. In cultured human TM cells, the upregulation of ANGPTL7 caused not only a decrease in fibronectin levels but also disrupted the normal organization of fibronectin in the matrix [47]. ANGPTL7 also appeared to regulate fibronectin levels in human organ-cultured anterior segments [47] since the knockdown of its expression resulted in higher levels of fibronectin expression both in the presence and absence of dexamethasone. This suggests that the upregulation of ANGPTL7 may be a negative regulator of fibronectin expression in the TM/SC.

Although TGF-β2 and glucocorticoids appear to be predominant factors that influence fibronectin expression in glaucoma, metabolites found in the aqueous humor may also contribute to the dysregulation of fibronectin synthesis [52]. In particular, high levels of ascorbic acid and glucose have been reported to increase fibronectin levels in cultures of TM cells [53,54,55]. High glucose levels are found in the aqueous humor of patients with diabetes who tend to experience higher frequencies of glaucoma. This suggests that patients with diabetes may have higher levels of fibronectin synthesis in the TM/SC. A direct correlation between fibronectin levels and ascorbic acid in modulating IOP has not been demonstrated and may depend on the specific derivative of ascorbic acid present since a recent metabolome study has shown that o-methylascorbate has a significant IOP-lowering effect [56].

Finally, components within the ECM may also influence fibronectin expression in the TM/SC. For instance, hyaluronan, which is a major component of the TM/SC, appears to regulate fibronectin expression in the outflow pathway. When hyaluronan synthesis is inhibited, cultured porcine TM cells express lower levels of fibronectin [57]. A similar decrease in fibronectin levels was observed in cultured human anterior segments treated with 4-methylumbelliferone, an inhibitor of hyaluronan synthesis [57]. This decrease in fibronectin expression is in contrast to that observed in lung myofibroblasts, where inhibiting hyaluronan synthesis with 4-methylumbelliferone enhanced fibronectin expression [58].

Whether the effect on fibronectin expression is specifically due to an interplay between hyaluronan synthesis and fibronectin or to the subsequent loss of other ECM components such as versican when hyaluronan synthesis is inhibited is not known. However, some studies have shown a more direct interaction. For instance, synthetic hyaluronan was found to increase fibronectin levels in differentiated human bone marrow stromal cells in culture, which further supports the observation that hyaluronan regulates fibronectin levels [59]. In addition, it also appears to affect fibril formation since the fibronectin fibrils assembled were thinner and their overall level deceased in the presence of the synthetic hyaluronan. This suggests that the function of fibronectin fibrils in the TM/SC could be altered if hyaluronan levels are changed. Interestingly, there are decreased levels of hyaluronan in the aging human eye and in POAG [60,61], which suggests that fibronectin activity could be altered under these circumstances.

### Isoforms of Fibronectin in the Trabecular Meshwork

**Figure 2:**
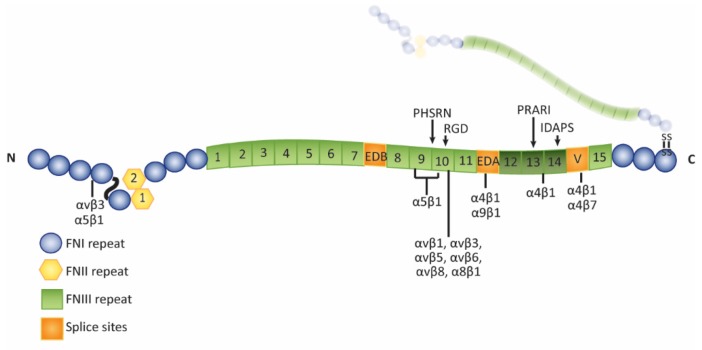
Diagram of fibronectin: Dimeric fibronectin consists of two nonidentical polypeptide chains that are connected by a disulfide bond. The chains are comprised of three modules called type I (blue circles), type II (yellow hexagons), and type III (green squares) repeats. Three regions are alternatively spliced (Extra Domain A, EDA; Extra Domain B, EDB; and V; orange squares) to give rise to the various isoforms of fibronectin. The cell binding domain (CBD) containing the RGD (Arg-Gly-Asp amino acids) motif is located in the 10th type III repeat. The HepII domain spans the 12th–14th type III repeats (darker green squares).

Fibronectin mRNA can undergo alternative splicing to create 20 isoforms of fibronectin [62]. During splicing, the exons for the EDA and EDB (extra domain B) domains, separately or together, can be spliced out to create EDA− and/or EDB− isoforms of fibronectin (Figure 2). The exon for the IIICS (type III connecting segment) region can also undergo splicing to generate 5 splice variants of this domain. Expression of these isoforms is age related and can change during development and pathological processes [63,64]. In adults, most fibronectin isoforms found in tissues lack both the EDA and EDB domains, but fibronectin isoforms containing these domains can be upregulated during times of tissue remodeling or diseased states. In normal human adult donor eyes, however, EDA+ fibronectin is localized within the TM/SC [21]. This suggests that, unlike other adult tissues, EDA+ fibronectin may be constitutively expressed in the TM/SC. Whether the EDB+ isoform of fibronectin is also present in vivo is unknown. In cultured human TM cells, both EDA+ fibronectin and low levels of EDB^+^ fibronectin are expressed under routine culture conditions [21,22].

The presence of EDA+ isoforms of fibronectin in the TM/SC region is not unexpected, given that aqueous humor contains growth factors such as TGF-β1 and TGF-β2 that can affect alternative splicing of fibronectin [16,20,21,22,65]. For instance, in cultured porcine TM cells [66], the EDA and EDB domains appear to be spliced out of fibronectin transcripts when cells are grown in the absence of serum which contains growth factors. However, when these cells are cultured in serum, a percentage of transcripts contain both the EDA and EDB exons. Similarly, cultured human TM cells grown in the presence of serum always appear to express some EDA+ and EDB+ fibronectin [21,22]. It is unknown whether in vivo expression of the EDA+ isoform of fibronectin in the TM/SC occurs as a result of aging or is always expressed in the TM/SC, since aging of TM cell cultures has been reported to cause an increase in the expression of fibronectin [13].

Mechanical stretching in vitro, which is thought to mimic the in vivo contraction and relaxation of the TM/SC region in response to normal fluctuations in IOP, does not affect splicing of fibronectin despite the fact that it is reported to affect alternative splicing of other ECM proteins in the TM [67,68]. In both stretched and unstretched porcine TM cell cultures grown under serum-free conditions, at least 90% of the fibronectin transcripts lacked the EDB exon and none of the transcripts contained the EDA exon [69].

Interestingly, aqueous humor contains growth factors known to affect alternative splicing of fibronectin, yet fibronectin found in the aqueous humor lacks both the EDA and EDB domains and this was unchanged in patients with POAG [15]. In both cataract patients and patients with POAG, EDA− and EDB− fibronectin is the predominant form of fibronectin found in aqueous humor. This differs from fibronectin found in the ECM of TM/SC tissues from patients with POAG in which EDA+ fibronectin was found [21]. This suggests that the soluble fibronectin found in aqueous humor within the anterior chamber does not come from TM cells. Whether fibronectin in aqueous humor from POAG patients contains the EDB domain is not known.

The inclusion of the EDA domain in fibronectin suggests that fibronectin could play a role in modulating aqueous humor outflow and IOP by regulating the expression of matrix metalloproteinases (MMPs) [70]. The ECM in the TM/SC region is not a static structure and appears to undergo remodeling as a way to maintain IOP homeostasis. The ECM remodeling arises mainly from the activity of MMP-2, MMP-3, and MMP-9, but other proteases such as cathepsin B and urokinase-type plasminogen may also be involved [71,72,73,74,75,76,77]. Interestingly, laser trabeculoplasty, a procedure performed to treat glaucoma, induces an increase in levels of MMPs that results in a remodeling of the ECM in the JCT region of the TM that leads to long-term reduction in IOP [78,79]. 

Alternately, expression of the EDA+ isoform of fibronectin could be modulating the contractile properties of the TM/SC. As mentioned above, the TM/SC is a highly contractile tissue that has smooth-muscle like features [80,81] that are regulated by RhoGTPase activity [82,83,84]. In POAG, it has been postulated that sustained activation of RhoA by TGF-β2 and/or CTGF signaling promotes the differentiation of TM cells into myofibroblasts [32,84]. In mice overexpressing CTGF, treatment with a Rho-kinase inhibitor interferes with actin contractility and causes a reduction in IOP that is comparable to normal mice. This demonstrates that the contractile properties of the tissue play an important role in mediating IOP [32,85].

This transition to the myofibroblast phenotype may be triggered in part by the increase in EDA+ fibronectin expression that is also caused by elevated levels of TGF-β2 in the aqueous humor [21]. Ultimately, this is believed to induce a myofibroblastic-like environment in the TM/SC which would impair the ability of the TM/SC cells to respond to sustained changes in IOP. The cellular responses mediated by the EDA domain can involve interactions with both integrins and the Toll-like receptor 4 (TLR4) [86,87,88]. Integrins that bind the EDA domain include α4β1, α4β7, and α9β1 [86,87,88,89,90,91]. The α4β1 integrin is expressed by TM cells in culture and in the TM of human eyes in vivo [92,93,94]. SC cells express α9β1 integrin [95]. To date, α4β7 integrin has not been reported in TM or SC cells. TLR4, which can also bind the EDA domain of fibronectin, is expressed in cultured human TM cells and in the TM of human and mouse eyes in vivo [96,97]. Thus, both TM and SC cells are likely to interact with the EDA domain of fibronectin and to respond to it by converting into a myofibroblastic-like phenotype. Interestingly, EDA+ fibronectin also plays a role in modulating TGF-β signaling, suggesting that a bidirectional signaling mechanism exists between fibronectin expression and TGF-β signaling [98]. Such a mechanism in the TM/SC could be important in maintaining TM/SC homeostasis.

## 3. How Fibronectin Affects IOP 

A key feature of fibronectin’s biological activity is its modular and flexible structure [64,99]. The major form of fibronectin in aqueous humor is plasma fibronectin, which lacks both the EDA and EDB domains. As a soluble protein in aqueous humor, fibronectin would exist as a compact protein with many of its biologically active domains inaccessible. The active form of fibronectin in the TM/SC, however, is most likely an extended protein assembled into an insoluble fibril. Little is known about the activity of fibronectin in the TM/SC and how it regulates outflow resistance. Extensive literature from other cell types suggests that fibronectin and its receptors could regulate many of the biological processes involved in modulating outflow resistance, including matrix production and turnover, gene expression, growth factor signaling, and cytoskeletal organization [100,101,102,103,104]. Fibronectin and its receptors also modulate cellular mechanoresponsiveness to physical forces such as stretch [105], which occurs when IOP is elevated.

### 3.1. Increased ECM Rigidity

In normal eyes, numerous studies have suggested that the anterior segment can sense changes in IOP and can respond by adjusting outflow resistance across the TM/SC [4,73,106,107,108]. Hence, when the pressure is elevated due to fluid buildup, the architecture of the TM/SC is thought to be temporally and spatially modified to allow greater outflow in order to reduce the pressure [109]. Conversely, when pressure is low, the resistance is increased to restrict outflow. Interestingly, these properties are thought to occur specifically within the JCT and SC but vary throughout the entire TM/SC [68] since some regions of the JCT and SC exhibit greater flow than other regions. Atomic force microscopy studies support the idea of “regions of segmented flow”, where the rate of aqueous humor outflow across the TM can vary significantly and can further demonstrate that these differences could be due to variations in the elastic modulus between these regions of high and low flow. Regions of high flow had a lower elastic modulus and thus appeared to be consistently more compliant than low-flow regions [110].

This suggests that the biomechanical properties of the cells and ECM within the JCT and SC modulate the drainage of aqueous humor through the anterior segment [68]. How the properties of cells and surrounding ECM contribute to regions of segmental outflow of aqueous humor is still being investigated. One thought has been that the composition of the ECM in these regions differs, thereby creating regions with different biomechanical properties. In support of this idea, it has been observed that the distribution of fibronectin as well as other ECM components such as versican and hyaluronan varies throughout the JCT and SC [12,111]. Although a correlation between fibronectin expression and regions of high and low flow has not been studied, upregulation of fibronectin expression has been shown to reduce TM cell monolayer permeability [112].

In glaucoma, the biomechanical properties of the TM/SC are altered. Glaucomatous tissues have a higher elastic modulus which is thought to be due to the increased expression of ECM proteins including fibronectin [35,113], enhanced cross-linking of the ECM by tissue transglutaminase [114,115] as well as changes in the expression of several matrix proteins along regions of high and low flow [110]. The increased elastic modulus or rigidity of the TM/SC is likely to affect fibronectin fibril formation and, ultimately, any cell-matrix signaling via fibronectin fibrils. In vitro, the assembly of fibronectin fibrils has been shown to be highly dependent on contractile forces generated by the actomyosin network and on the stiffness of the substrate. On stiff substrates, fibronectin fibrils in TM cultures appear as an elaborate network of long fibrils [116], but on soft substrates, the fibrils appear shorter and as individual unbranched fibrils. 

**Figure 3:**
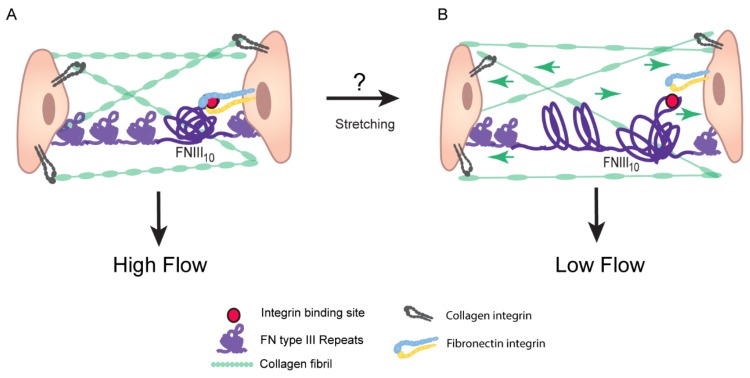
Contractile forces of the TM/SC can alter flexibility of fibronectin fibrils and integrin signaling: (**A**) When the trabecular meshwork extracellular matrix is relaxed, the tertiary structure of each fibronectin type III repeat would be expected to be folded into individual β-sandwich structures [64]. In the case of the 9th and 10th type repeats, this conformation places the synergy and RGD sequence closer together, thus creating a high-affinity binding site for the α5β1 integrin [123]. (**B**) As the extracellular meshwork is stretched, perhaps due to increased IOP or the contractile forces of the ciliary muscle and TM and SC cells, these fibronectin type III repeats would be unraveled [128]. Unraveling of the 9th and 10th type III repeats would increase the distance between the synergy and RGD binding sites in the 9th and 10th repeats respectively, potentially disrupting binding between fibronectin and the α5β1 integrin. These differences in integrin signaling events could explain why regions of high and low flow of aqueous humor across the TM exist.

The rigidity of the ECM, which is altered in POAG patients [35] and in glaucomatous cell cultures [117], is likely to affect the structure and activity of fibronectin fibrils. The type III repeats in fibronectin lack intrachain disulfide bonds which enables cell contraction to stretch these repeats and to expose cryptic sites with unique biological activities. One example of this is the FN III1 repeat. Applying a 30–35% stretch to immobilized fibronectin exposes a cryptic site in the FN III1 repeat that promotes the assembly of fibronectin fibrils [118]. Importantly, the extensibility of these type III repeats is retained even after fibronectin has been assembled into a fibril [99] such that a fibronectin fibril can be stretched about four times its original length [119]. The force required to stretch fibronectin fibrils can be generated by the actomyosin network [119,120] and can lead to a complete loss of the quaternary structure of fibronectin. This suggests that changes in the contractile properties of the trabecular meshwork due to enhanced rigidity of the TM/SC or elevated IOP would affect stretching of fibronectin fibrils and hence its biological activity. For instance, stretching the fibronectin fibril could affect binding to the α5β1 integrin, which plays an important role in regulating the contractile forces of the cells [121]. High affinity binding of α5β1 integrin to fibronectin requires interactions with both the RGD (Arg-Gly-Asp amino acid sequence) and synergy sites in FN III10 and FN III9, respectively. When the fibril is stretched, the distance between these sites increases which prohibits α5β1 integrin from binding both sites (Figure 2 and Figure 3) [122,123,124], thus altering signaling from α5β1 integrin. Stretching of fibronectin fibrils may also change the 3-D architecture of the ECM because it could alter the binding sites used by fibronectin-binding proteins such as tenascin-C [125], CD44 [126], versican [127], and myocilin [39].

### 3.2. Fibronectin Regulates Deposition of Collagen IV, Laminin, and Fibrillin

As a major component of the ECM, fibronectin fibrils play a critical role in the formation of the ECM, are known to control aqueous humor outflow, and contribute to the pathogenesis of glaucoma. The elevation in IOP is generally attributed to a reduction in aqueous humor outflow through the trabecular meshwork as a result of excessive TM extracellular matrix (ECM) production and/or decreased turnover [7,108,129]. Fibronectin is one of the earliest ECM fibrils to be assembled into the ECM [64]. These fibrils mediate the incorporation of other proteins into the ECM by acting as a scaffold or organizing nidus upon which additional ECM proteins like collagen types I and III and fibrillin are assembled into the matrix [130,131,132,133,134].

The ECM of the TM is unique in that the basement membrane also contains fibronectin as part of that network in addition to laminin and type IV collagen [9,135]. The TM/SC also contains an elastin fiber system containing elastin, type IV collagen, and fibrillin-1 in the uveal and JCT regions of the TM and along the inner walls of Schlemm’s canal [136]. When fibronectin fibril formation in TM cell cultures was inhibited using a peptide derived from the *Streptococcus pyogenes* Functional Upstream Domain (FUD) of the F1 adhesion protein FUD [137], the de novo assembly of type IV collagen, laminin, and fibrillin into nascent matrices was also inhibited [22]. It is not clear how fibronectin fibrils would promote this type IV collagen/laminin network formation. No specific binding site(s) within fibronectin has been identified for laminin or type IV collagen, however, direct binding between fibronectin and type IV collagen has been reported [138]. Evidence of codependence between matrices of fibronectin and type IV collagen has also been reported in earlier studies. For example, co-localization of type IV collagen and fibronectin fibrils has been reported in endothelial cells [139] and fibroblast cultures [140]. Additionally, in Schwann cells [141], fibronectin fibrillogenesis was dependent on the presence of type IV collagen. The interrelationship between the formation of type IV collagen and laminin matrices has been well established [142]. Thus, it is possible that if the type IV collagen network fails to form when fibronectin fibrillogenesis is blocked by FUD, the stability of the laminin network is also impacted.

Interestingly, while FUD was still effective in promoting the removal of fibronectin fibrils from established fibronectin matrices, it had no effect on mature matrices of type IV collagen, laminin, and fibrillin [22]. Thus, while fibronectin fibrils were required for the development of nascent matrices of type IV collagen, laminin, and fibrillin, this does not appear to be the case for mature matrices of the same proteins. This was not unexpected since both fibrillin and the laminin/type IV collagen network are usually considered to be separate structural entities once they are assembled [142,143].

### 3.3. Bioactivity of HepII Domain Affects IOP

Fibronectin is a multi-domain protein with each domain exhibiting a remarkable number of biological activities [64]. Many of the domains are proteolytically resistant and can be isolated without loss of activity. This means that small bioactive domains of fibronectin would be available in the TM in vivo when the normal turnover of ECM occurs and thus could play a role in regulating IOP. One such domain that is relevant to the regulation of outflow resistance and IOP is the HepII domain of fibronectin. This domain consists of the 12th–14th type III repeats (Figure 2) and can bind the glycosaminoglycan (GAG) side chains of heparan sulfate proteoglycans (HSPGs) which are found on the cell surface of TM cells [93] and in the ECM of the TM [4,144,145]. The HepII domain has also been reported to contain a binding site for α4β1 integrin [146].

In vitro studies showed that when this fragment of fibronectin was perfused into organ-cultured human and monkey anterior segments, the movement of fluid through the anterior segment (outflow facility) was increased by 93% and IOP was significantly decreased [147,148]. A similar finding has also been observed in porcine organ-cultured anterior segments (unpublished data). How the HepII domain increases outflow is still unclear, but in vitro studies using cultures of human TM cells suggested that the HepII domain disrupted the actomyosin cytoskeleton and decreased the contractile properties of the cells [149]. These studies showed that the HepII domain-mediated disruption of the cytoskeleton depended upon the presence of type IV collagen in the ECM and involved the α4β1 integrin and the PRARRI sequence within the HepII domain. siRNA silencing of the expression of the syndecan-4 HSPG or removal of cell surface heparan sulfate by heparitinase treatment did not prevent the HepII domain-mediated disruption of the actin cytoskeleton [94]. Thus, the influence of the HepII domain on IOP homeostasis could involve signaling between α4β1 integrin and, possibly, the collagen-binding integrins α1β1 and/or α2β1.

The HepII domain also binds myocilin [39,150], a glucocorticoid response protein associated with glaucoma, and vascular endothelial growth factor (VEGF) [151], which is present in aqueous humor [152]. Myocilin has been shown to impair the incorporation of paxillin into focal adhesions [150]. VEGF can regulate MMP activity in the TM [153] and hence endothelial cell permeability [154]. Thus, the HepII domain could be an important matrix reservoir for VEGF and myocilin. When the HepII domain is released during turnover of the ECM, there could be a subsequent release of VEGF and/or myocilin. This, in turn, could increase the permeability of the TM/SC outflow pathway. Myocilin could disrupt stress fiber assembly in TM cells, thereby enhancing outflow facility via a relaxation of the TM/SC. The increase in unbound VEGF, which has been shown to increase outflow facility in ex vivo mouse eye cultures [155], would increase the permeability of the endothelium of Schlemm’s canal [156] and possibly control the localized activity of MMPs reported to enhance outflow facility [73,74].

## 4. Assembly of Fibronectin Fibrils

The assembly of fibronectin into fibrils is a highly regulated integrin-mediated process [157,158,159]. Once bound to the integrin, the tertiary and quaternary structures of the fibronectin dimer are altered, thereby exposing numerous fibronectin-fibronectin binding sites needed to promote the lateral and linear association of fibronectin dimers into a fibril. As in other cell types, the α5β1 integrin appears to act as the primary receptor that promotes fibril formation in TM cell cultures [159,160] and interactions with the amino terminus of fibronectin are critical for fibril formation [22,160,161]. However, studies have also indicated that the αvβ3 integrin can participate in fibril formation in fibroblasts [162] and TM cells [160].

αvβ3 integrin is found on both TM and SC cells [22,94,163]. Earlier studies on αvβ3 integrin signaling in TM cells in culture indicated that TGF-β2 and dexamethasone can induce αvβ3 integrin signaling [164,165] and that activation of this integrin signaling mimics the glaucomatous phenotype observed in cultured cells and in vivo [22]. Among the changes induced by the activation of αvβ3 integrin is an increase in fibronectin fibril formation that is observed in TGF-β2 and glucocorticoid-induced models of ocular hypertension or cultures treated with TGF-β2 or dexamethasone [21,22,40,41,45,166,167,168].

A recent study [160] found that overexpression of a constitutively active αvβ3 integrin in immortalized TM cells resulted in an increase in fibronectin fibrillogenesis and deposition into the ECM. This did not occur when wild-type αvβ3 integrin was overexpressed in these cells. This enhanced fibrillogenesis was not due to αvβ3 integrin replacing the role of α5β1 integrin in fibril formation as the initial stages of fibril formation could still be inhibited with a β1 integrin function-blocking antibody. Additionally, α5β1 integrin, but not αvβ3 integrin, was found in fibrillar adhesions, which are sites of fibronectin fibril formation. The increased fibrillogenesis could reflect the enhanced RhoA activity observed in the cells overexpressing constitutively active αvβ3 integrin. It is well established that fibronectin fibrillogenesis is a RhoA-dependent process, and increased RhoA activity in fibronectin fibril formation has been reported elsewhere [118,169,170,171]. The enhanced RhoA activity influences the contractile state and traction force generated by a cell through sites of focal adhesions, which in turn would promote the unfolding and stretching of the fibronectin dimer and subsequent fibrillogenesis. Interestingly, the enhanced fibrillogenesis in TM cells in response to activated αvβ3 integrin appears to utilize an alternative Rho kinase (ROCK)-independent pathway possibly involving RhoA/mDia [121]. Whether increased fibronectin fibril formation is responsible for the elevated levels of IOP observed in glaucoma is unclear at this time. However, increases in fibronectin fibril formation are frequently observed in TGF-β2-induced models of ocular hypertension or cultures treated with TGF-β2 [20,21,45,167]. An increase in fibronectin is also often observed in the TM following treatments with dexamethasone in vivo and in TM cultures [22,40,166,168]. Thus, it is part of the phenotype reported to be associated with glaucoma.

### Fibronectin Fibrils Have Different Conformation and Composition

Interestingly, the composition and stretched state of fibronectin in fibrils appeared to be altered when the αvβ3 integrin is activated [160]. Studies found that the fibronectin fibrils in cells overexpressing a constitutively activated αvβ3 integrin contain substantially more EDA+ and/or EDB+ fibronectin than fibrils assembled by cells expressing low levels of αvβ3 integrin or overexpressing wild-type αvβ3 integrin. This change in fibril composition was not due to the levels of fibronectin changing since the amount of EDA+ fibronectin expressed was similar regardless of the levels or activation state of the integrin. This suggests that activation of αvβ3 integrin is involved in promoting the assembly of fibronectin fibrils that include one or both of these domains.

Studies have shown that matrices can contain a mixture of fibronectin fibrils with different conformations. Using fluorescence resonance energy transfer (FRET) analysis to detect the conformation of fibronectin in matrices, fibronectin in fibrils exhibited a highly extended conformation compared to fibronectin bound to the cell surface [172]. Furthermore, the fibronectin within matrices exhibited varying degrees of unfolding, suggesting that fibronectin fibrils with different conformations can coexist within the same matrix. This supports earlier high resolution Cryo-SEM studies [173] on fibronectin fibrils formed in vitro that found that some fibrils were very straight whereas others were highly nodular and coiled. Additionally, these studies showed that epitopes found within the HepII domain of fibronectin, which has been found to lower IOP in eye organ cultures studies [147], were only detected in the straight fibrils. This suggests that, under high IOP conditions where the fibronectin fibrils within the TM/SC are more likely to be stretched, fibronectin domains like the HepII domain that appear to have outflow regulatory roles would be exposed within the matrix.

Recent studies in TM cell cultures support this idea that fibrils in the TM/SC may have different conformations. These studies found that, under conditions where αvβ3 integrin is activated [160], a higher percentage of fibrils are positively labeled with antibody mAb L8, which detects an epitope that is exposed when fibronectin is stretched in response to tension [118]. This suggests that fibronectin in fibrils assembled by cells expressing an activated αvβ3 integrin may be more unfolded [172]. This enhanced unfolding of fibronectin fibrils is associated with more rigid fibrils in aging matrices and would be expected to have different biochemical properties that would affect cell behavior [122]. The observation that EDB+ fibronectin is more prevalent in fibrils assembled by cells expressing activated αvβ3 integrin further substantiates this idea that fibrils assembled when αvβ3 integrin is activated may have different biological properties.

Recent studies by others support this and have suggested that inclusion of the EDB domain in fibronectin enhances VEGF expression and phagocytosis [174,175,176]. The enhancement in VEGF expression and phagocytosis was believed to be due to the exposure of sequences at the inter-domain interface between the EDB domain and the FN III 8 repeat of fibronectin. The enhancement in phagocytosis was also reported to be directly mediated by αvβ3 integrin. Together, these studies suggest that changes in the expression of different isoforms of fibronectin by glucocorticoids and TGF-β2 may result in the assembly of a fibronectin matrix with biological properties that differs from a matrix assembled under conditions where EDB+ fibronectin is either expressed at very low levels or not at all. Whether these changes could contribute to the enhanced rigidity of the ECM associated with glaucoma is unknown.

## 5. Concluding Remarks

POAG remains a poorly understood disease, and fibronectin is likely to play a multifactorial role in the TM/SC in glaucoma. The dysregulation of its expression in POAG including alternative splicing, matrix assembly, expression levels, and turnover can affect how the TM/SC responds to fluctuations in IOP. Studies evaluating the pathways and role of metabolites in the regulation of fibronectin synthesis and turnover are needed to advance our understanding of the pathogenesis of POAG and to develop new therapeutic targets for POAG.
